# Supplementary material for: Ammonium Glycyrrhizinate Prevents Apoptosis and Mitochondrial Dysfunction Induced by High Glucose in SH-SY5Y Cell Line and Counteracts Neuropathic Pain in Streptozotocin-Induced Diabetic Mice
Source: Biomedicines. 2021 May 26;9(6):608. doi: 10.3390/biomedicines9060608 (PMC8227813; doi:10.3390/biomedicines9060608)
Supplement: Supplementary file 1 [file biomedicines-09-00608-s001.zip › biomedicines-1218104-supplementary.pdf]

biomedicines

MDPI

Article

Ammonium Glycyrrhizinate Prevents Apoptosis and Mitochondrial Dysfunction Induced by High Glucose in SH-SY5Y Cell Line and Counteracts Neuropathic Pain in Streptozotocin-Induced Diabetic Mice

Laura Ciarlo [1], Francesca Marzoli [1], Paola Minosi [1], Paola Matarrese [2*] and Stefano Pieretti [1*]

[1] National Center for Drug Research and Evaluation, Istituto Superiore di Sanità, viale Regina Elena 299, 00161, Rome, Italy; laura.ciarlo@iss.it (L.C.); francesca.marzoli@iss.it (F.M.); paola.minosi@iss.it (P.M.)
[2] National Center for Gender-Specific Medicine, Istituto Superiore di Sanità, Viale regina Elena 299, 00161, Rome, Italy; paola.matarrese@iss.it
* Correspondence: paola.matarrese@iss.it, (P.M.); stefano.pieretti@iss.it (S.P.)
† These Authors contributed equally to this work.

Supplementary materials

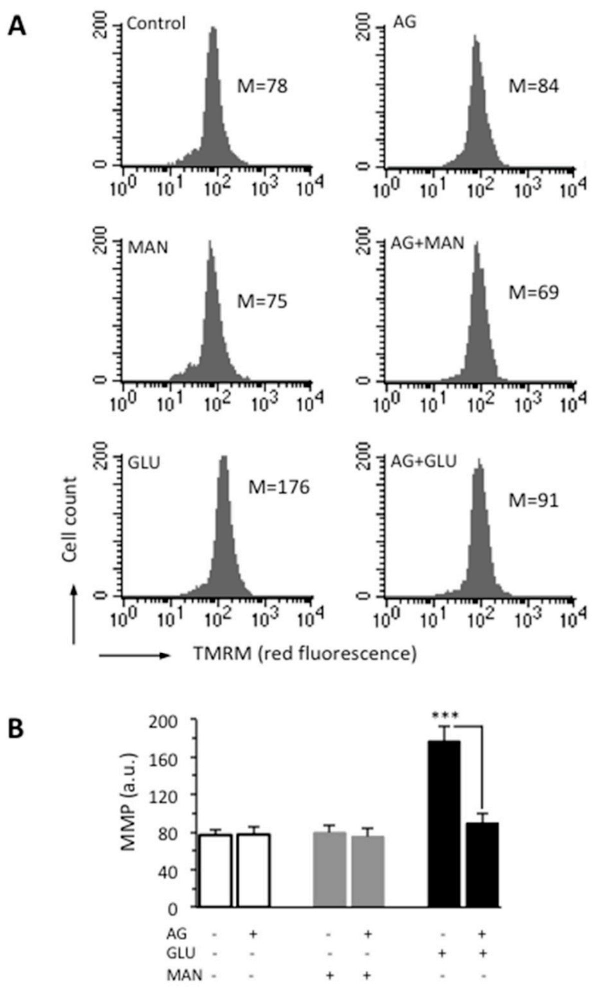

Figure S1
